# ﻿*Colocasia sookchaloemiae* (Araceae), a new species from northeastern Thailand

**DOI:** 10.3897/phytokeys.269.175203

**Published:** 2026-01-13

**Authors:** Wilawan Promprom, Phukphon Munglue, Pattana Pasorn, Soulivanh Lanorsavanh, Wannachai Chatan

**Affiliations:** 1 Department of Biology, Faculty of Science, Mahasarakham University, Kantharawichai District, Maha Sarakham 44150, Thailand; 2 Plant and Innovation Research Unit, Mahasarakham University, Maha Sarakham 44150, Thailand; 3 Program of Biology, Faculty of Science, Ubon Ratchathani Rajabhat University, Ubon Ratchathani 34000, Thailand; 4 Walai Rukhavej Botanical Research Institute, Mahasarakham University, Maha Sarakham 44150, Thailand; 5 Department of Biology, Faculty of Natural Sciences, National University of Laos, Vientiane, Laos

**Keywords:** Asia, new species, taxonomy

## Abstract

*Colocasia sookchaloemiae* (Araceae) is described from Phu Pha Yol National Park (northeastern Thailand). It is morphologically similar to *C. fallax*, from which it differs in having seasonal dormancy (vs. evergreen), broader and non-glaucous laminae (vs. narrower and slightly glaucous beneath), a speckled green ovary (vs. mostly green), a convex stigma with a depressed center (vs. 3-lobed), a sparsely hairy sterile interstice (vs. glabrous), and a thicker stipitate appendix (vs. slender, often sessile, or shortly stipitate). A comparative study of living plants, cultivated specimens, and herbarium material (including types) supports its recognition as a distinct species. The new taxon grows on rocky cliffs in dry dipterocarp and mixed deciduous forests at 350–400 m a.s.l., and it is currently known from Mukdahan and Sakon Nakhon provinces. A preliminary IUCN assessment indicates that the species is Data Deficient (DD).

## ﻿Introduction

The genus *Colocasia* Schott (family Araceae Juss., tribe Colocasieae Engler) comprises 14 accepted Asian species, naturally distributed in South and Southeast Asia, China, and Taiwan; some taxa are considered alien on other continents (POWO 2025), especially *Colocasia esculenta* (L.) Schott (see e.g., Iamonico 2021; Guzzon et al. 2025). Members of *Colocasia* are perennial herbs typically growing in moist to wet environments, including rainforests, riverbanks, and swamps; they can be distinguished by their large sagittate or peltate leaves and are valued for ecological functions as well as cultural, ornamental, and agricultural purposes. Among them, *C. esculenta* (taro) is of particular economic importance, being extensively cultivated for its edible corms (Guzzon et al. 2025). The work by Sangnin and Sookchaloem (2008) was among the earliest efforts to synthesize knowledge of *Colocasia* in Thailand and highlighted morphological variability within and between species that may reflect unrecognized taxa. Currently, the Flora of Thailand includes three species, i.e., *C. esculenta*, *C. fallax* Schott, and *C. menglaensis* J.T.Yin, H.Li & Z.F.Xu (see Boyce and Sookchaloem 2012; POWO 2025).

In recent years, discoveries of new species and records of Araceae in Phu Pha Yol National Park have underscored the remarkable yet incompletely explored diversity of this region. Notable taxa include *Alocasia sakonakhonensis* Chatan & Promprom, *Amorphophallus coudercii* (Bogner) Bogner, and *A. sakonnakhonensis* Chatan & Promprom (Promprom and Chatan 2023; Promprom et al. 2023; Promprom et al. 2024). The discovery and documentation of new aroid species, together with new distributional records from Thailand, indicate that this group is both taxonomically diverse and potentially cryptic, with certain taxa distinguished only by subtle morphological or reproductive traits.

During fieldwork carried out in Phu Pha Yol National Park between 2023 and 2024, we found an unidentifiable population of *Colocasia*. Careful examination of living material in the field, combined with comparative study of herbarium specimens and taxonomic investigation, confirmed that this species possesses a unique set of morphological traits that clearly separate it from all previously described congeners. Consequently, we here describe this taxon as a new species.

## ﻿Materials and methods

Plant material was collected during field surveys in Mukdahan and Sakon Nakhon Provinces during the period 2023–2024. Morphological observations of the new species were carried out on living plants as well as on herbarium specimens preserved at BK and BKF (codes according to Thiers 2025). Some specimens were cultivated in the authors’ home garden for further morphological observation. Measurements were made using a vernier caliper or an ocular micrometer in a dissecting microscope.

Relevant literature (e.g., Gagnepain 1942; Li and Boyce 2010; Boyce and Sookchaloem 2012; Zhou et al. 2020) was also analyzed. In addition, digital images of herbarium specimens—including the type specimen of the most similar species, *Colocasia fallax* (Hooker, J.D.H. & Thompson, T., 1904; K000499479 [K])—deposited in various international herbaria were obtained through JSTOR Global Plants (https://plants.jstor.org/) and individual herbarium websites, and these were carefully examined alongside the collected material.

The preliminary conservation status of the species was evaluated following the criteria and guidelines of the IUCN Standards and Petitions Committee (2024) to provide an initial assessment of its risk of extinction and conservation priority.

## ﻿Results

### ﻿Taxonomic treatment

#### ﻿ Colocasia sookchaloemiae

Taxon classificationPlantaeAlismatalesAraceae

Chatan & Promprom sp. nov.

D361F707-6520-5949-8FDE-EEA77748D3AA

urn:lsid:ipni.org:names:77374961-1

Figs 1, 2, 3, 4, 5

##### Type.

Thailand • Mukdahan Provinces. 16°43'08.4"N, 104°25'24.1"E, alt. ca. 400 m, 30 August 2023, *W. Chatan 2472* (holotype, BKF!; isotype: BK!).

##### Diagnosis.

*Colocasia sookchaloemiae* differs from *C. fallax* in having seasonal dormancy (*vs.* evergreen); broader (11–25 cm) and non-glaucous lamina [*vs.* narrower (3.5–15 cm) and slightly glaucous beneath]; larger (2.3–3.1 mm in diameter), speckled green ovary and a convex stigma with a depressed center [*vs.* smaller ovary (ca. 1.1 mm in diameter), stigma 3-lobed]; pistillate zone with basal rows of pistillodes (*vs.* rows of nail-/peg-like staminodes); sterile interstice and staminate zone sparsely hairy (*vs.* glabrous); sterile interstice shape cylindrical and slightly constricted (*vs.* tapering); appendix thicker, ivory, and clearly stipitate (*vs.* slender, often sessile/shortly stipitate, creamy-yellow to purple). Details of the morphological comparison between the new species and *C. fallax* are shown in Table 1.

**Table 1.** Comparison of *Colocasia sookchaloemiae* and *C. fallax*.

	*Colocasia sookchaloemiae*	*Colocasia fallax*
**Habit**	Herb with seasonal dormancy	Evergreen herb
**Rhizome/Stem**	Rhizomes depressed-globose (1.2–1.8 × 1.8–2.2 cm) or elongate (1–5 cm long, 1–1.5 cm diam.)	Rhizome erect, up to 7 × 1–1.5 cm
**Stolons**	Up to 40 cm long, 3–4 mm diam.	Up to 45 cm long, ca. 3 mm diam.
**Petiole**	23–63 cm long, sheath 5–15 cm long, greenish, glossy	12–57 cm long, sheathing almost ¹/2 length, pale green to reddish brown sheath
**Leaf blade**	Broadly ovate to slightly circular, not glaucous, 15–35 × 11–25 cm, base cordate; adaxial side dull green, abaxial side pale green	Narrowly oblong-ovate cordate, 8–25 × 3.5–15 cm; adaxial side pale to mid-green, sometimes patterned; abaxial side slightly glaucous
**Peduncle**	18–22 cm	6.5–20 cm
**Spathe**	7.3–7.8 cm	6.5–16 cm long
**Lower part of spathe**	Elliptic or ovate, 1.8–2.2 × 1.0–1.2 cm, pale green	Ellipsoid-globose, 1.5–4 × ca. 1.3 cm, medium-green
**Spathe limb**	Broadly ovate, ca. 5.5 × 2.5 cm; abaxial side dull greenish yellow or white with a faint tinge of yellow; adaxial surface mainly yellowish green, becoming clearly dirty yellow to pale orange-yellow near the apex	Narrowly lanceolate, 5–12 × 1–3.5 cm, dirty yellow to pale orange-yellow
**Spadix length**	shorter than spathe	shorter than or equal to spathe
**Pistillate flower zone**	with 3–4 rows of white to ivory, globose to broadly ovate pistillodes at base	with 4–6 rows of whitish nail- to peg-like staminodes
**Ovary**	Broadly to very broadly ovate, 2.3–3.1 mm diam., speckled green	Subglobose, ca. 1.1 mm diam., green, sometimes speckled white
**Stigma**	Apex convex with depressed center	3-lobed
**Sterile interstice**	Cylindrical, 10–14 mm long, constricted in middle, both ends slightly expanded, sparsely hairy; lower part with weakly clavate to ovate staminodes; middle and upper parts with rhombohexagonal synandrodes	Tapering, 17–25 mm long, lower part with clavate staminodes, upper with rhombohexagonal synandrodes
**Staminate flower zone**	18–23 × 6–8 mm, sparsely hairy	11–14 mm × ca. 4 mm, hair absent
**Synandria**	3.0–5.2 mm diam., white to ivory	Ca. 1 mm diam., white to pale orange
**Appendix**	3.0–3.8 cm, 7–8 mm (~¹/3 spadix length), stipitate, ivory	2.5–3.5 cm × 1–3 mm (¹/2–¹/3 spadix length), sessile or shortly stipitate, creamy yellow to purple

##### Description.

Terrestrial, perennial herbs with a seasonal dormancy period, forming colonies or sometimes solitary among rocks, 30–45 cm tall. Rhizomes depressed-globose (1.2–1.8 × 1.8–2.2 cm) or elongate (1–5 cm long and 1–1.5 cm in diameter), with numerous stolons up to 40 cm long and 3–4 mm in diameter. Leaves always 2; petiole cylindrical, greenish, glossy, 23–63 cm long and 0.5–1.3 cm in diameter; sheath 5–15 cm long and 0.8–2.0 cm in diameter; leaf blade broadly ovate to slightly circular, peltate, 15–35 cm long and 11–25 cm wide; apex acuminate; base cordate. Upper surface slightly dull green; lower surface dull green, not glaucous. Primary veins palmate at the lamina base, with 2–3 pairs of primary lateral veins pinnately arranged toward the lamina apex, raised on the lower surface; midrib, lateral veins, and other veins level with the epidermal cells; primary lateral veins well demarcated; interprimary veins forming a weak interprimary collecting vein. Inflorescences 1–3 together, 20–30 cm long; peduncle cylindrical, pale green, dull, 18–22 cm long. Spathe constricted at about the lower third; the lower part (below the constriction) convolute, forming an elliptic or ovate organ, pale green, 1.8–2.2 cm long, and 1.0–1.2 cm in diameter. Limb broadly ovate, erect during the early blooming period, later fully spreading and folding downward at anthesis, ca. 5.5 × 2.5 cm, apex acuminate, dull greenish yellow or white with a faint tinge of yellow; adaxial surface mainly yellowish green, becoming clearly dirty yellow to pale orange-yellow near the apex; coloration faint or absent on the abaxial surface. Spadix 6–9 cm long, shorter than the spathe. Pistillate flower zone 1.0–1.2 cm long and 0.5–0.6 cm in diameter, with 3–4 rows of white to ivory, globose to broadly ovate pistillodes at the base. Ovaries broadly to very broadly ovate, 2.3–3.1 mm in diameter and 2.0–2.2 mm high, speckled green, not interspersed with staminodes, unilocular; ovules numerous, fusiform, translucent; placentae 3–4, parietal; stylar region short to absent; stigma apex convex with a depressed center, rod to broadly ovate pistillodes present at the end of this zone. Sterile interstice cylindrical, constricted at the middle with both ends slightly expanded; sparsely hairy, with soft white hairs becoming more distinct at maturity; 10–14 mm long and 1.7–2.6 mm in diameter. Lower part of sterile interstice with weakly clavate to ovate staminodes; middle and upper parts with rhombohexagonal synandrodes; entire interstice white to ivory. Flowers unisexual; perigone absent. Staminate flower zone white to ivory, 18–23 mm long and 6–8 mm in diameter, sparsely hairy with soft white hairs, becoming more distinct at maturity; synandria 3.0–5.2 mm in diameter, white to ivory. Appendix 3.0–3.8 mm long and 7–8 mm in diameter (ca. ¹/3 the length of the spadix), tapering cylindrical, with a rough surface. Distinctly stipitate, with a zone of 2–3 rows of staminodes at the base; sparsely hairy, with soft white hairs becoming more distinct at maturity. Male flowers 3–4-androus; stamens connate at the slightly grooved apex of the synandrium; thecae lateral, cylindrical, dehiscing by irregular rupture. Berry depressed-globose to irregular in shape, 5 mm in diameter, yellow. Seed solitary, ovoid to ellipsoid, ca. 2 mm in diameter, enclosed in a pale brown, translucent aril. Fruiting peduncle reflexed; fruiting spathe ovoid, ca. 2.5 × 1 cm, emerging as the leaves unfold.

**Figure 1.**
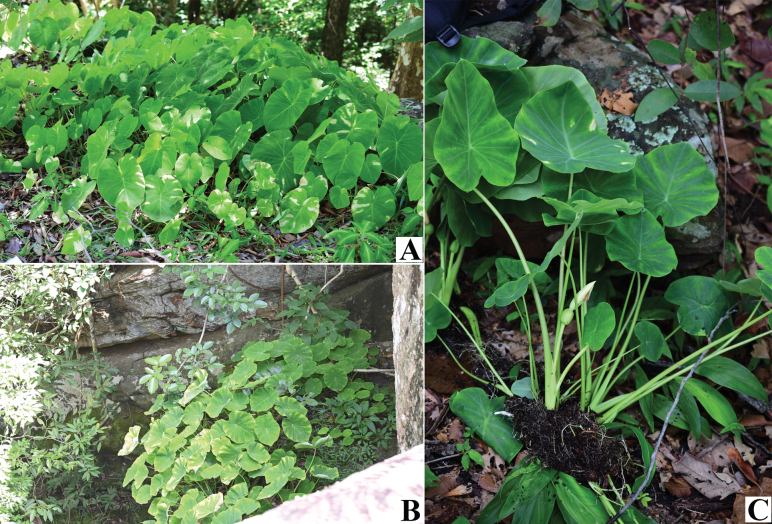
*Colocasia sookchaloemiae*. **A–C.** Habit and habitat.

**Figure 2.**
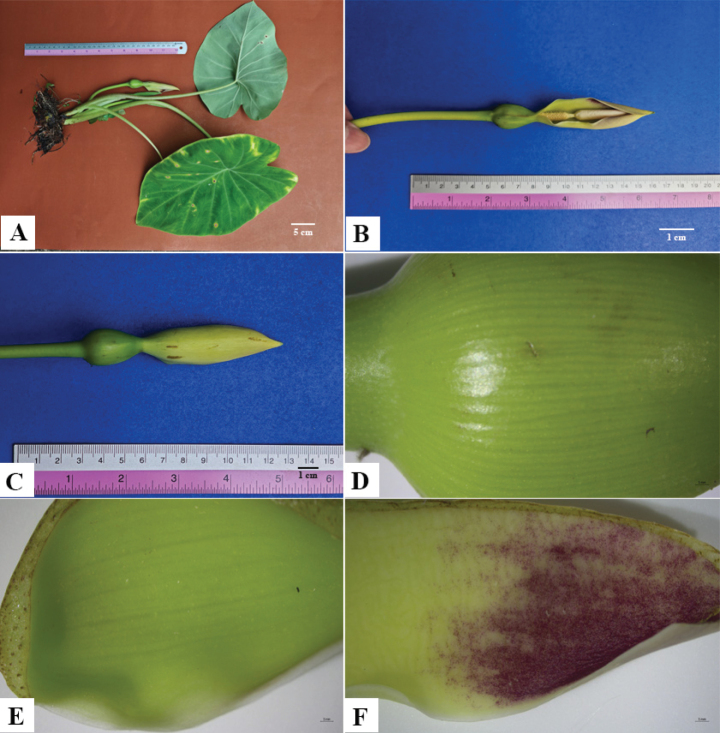
*Colocasia sookchaloemiae*. **A.** Whole plant; **B, C.** Inflorescence from different sides; **D.** Abaxial side of the spathe; **E, F.** Adaxial side of the spathe (lower and upper parts, respectively).

**Figure 3.**
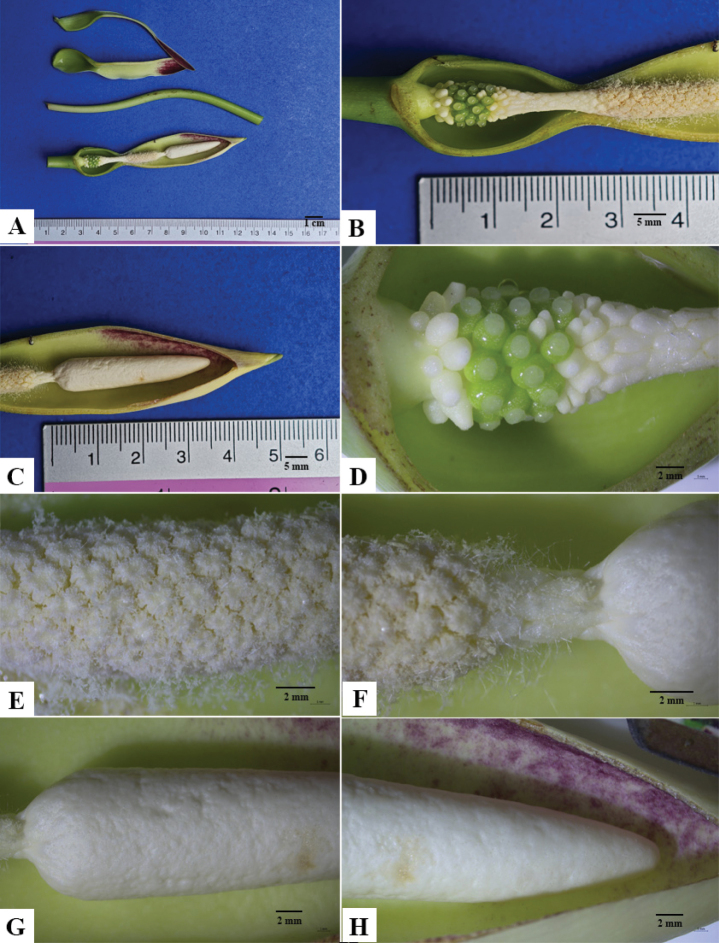
*Colocasia sookchaloemiae*. **A.** Opened inflorescence with parts removed; **B.** Lower part of the spadix showing pistillodes at the base, pistillate flower zone, sterile interstice, and part of the staminate zone; **C.** Upper part of the spadix and appendix; **D.** Enlarged view of the lower part of the spadix; **E, F.** Staminate flower zone, stipe, and lower part of the appendix; **G, H.** Appendix and adaxial side of the spathe (showing dirty yellow to pale orange-yellow coloration near the apex).

**Figure 4.**
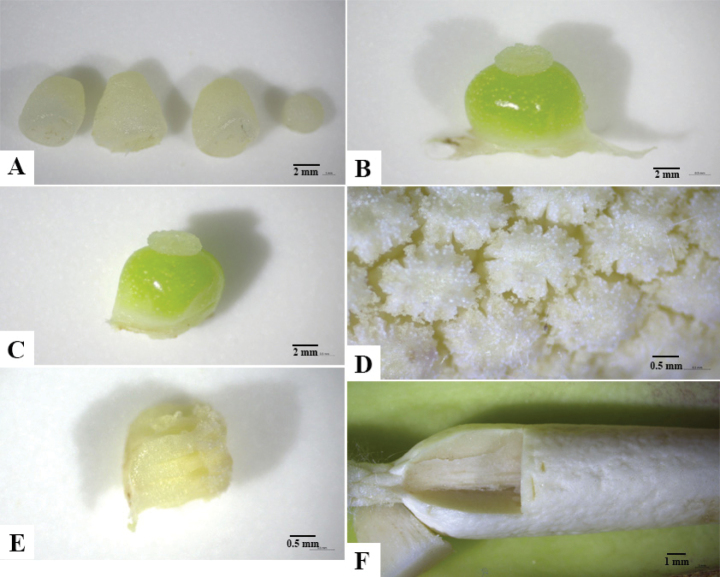
*Colocasia sookchaloemiae*. **A.** Pistillodes; **B, C.** Pistils (lateral views); **D, E.** Staminate flowers (top and lateral views, respectively); **F.** Appendix showing inner coloration.

**Figure 5.**
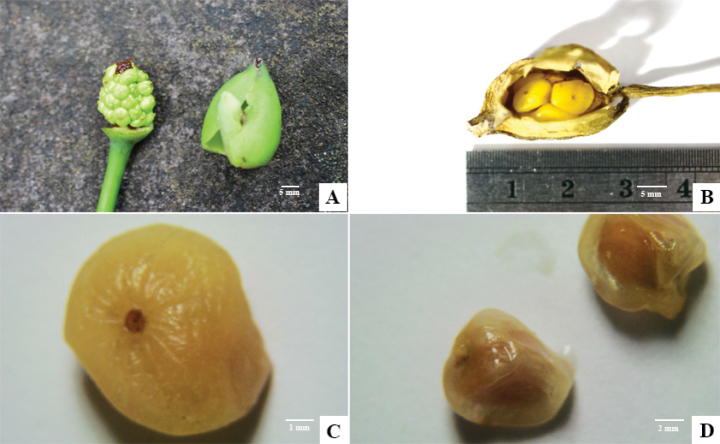
*Colocasia sookchaloemiae*. **A.** Young infructescence with removed spathe; **B.** Mature infructescence; **C, D.** Mature fruits.

##### Etymology.

The specific epithet honors Assoc. Prof. Dr. Duangchai Sookchaloem, an expert in aroid taxonomy in Thailand. She has devoted her career to the study and taxonomy of the family Araceae.

##### Phenology.

Flowering time June to July; fruiting time (mature fruits) October to December.

##### Distribution and habitat.

**Figure 6.**
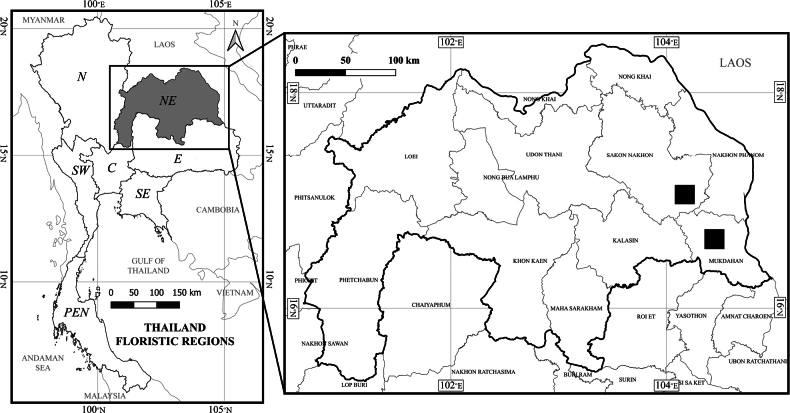
Distribution map of *Colocasia sookchaloemiae* in Mukdahan and Sakon Nakhon Provinces, northeastern Thailand (black square).

*Colocasia sookchaloemiae* is currently known from two populations occurring in Mukdahan and Sakon Nakhon Provinces, at elevations of 350–400 m. Nakhon Phanom Province is also expected to be part of its distribution range, as it is situated near the type locality and lies within the same protected area. The species grows on rocks and cliffs in dry dipterocarp and mixed deciduous forests at elevations of 350–400 m (Fig. 6).

##### Preliminary conservation status.

The preliminary conservation assessment, applying the IUCN criteria (IUCN Standards and Petitions Committee 2024), suggests that the species should be assigned to the Data Deficient (DD) category. In our opinion, the current data on population size, distribution, and habitat trends are insufficient to accurately evaluate its risk category. Further field surveys and long-term monitoring are necessary to assess population dynamics and potential threats, such as forest disturbance, tourism development, and climate variability in northeastern Thailand. Documenting and protecting narrowly distributed species remain crucial not only for biodiversity conservation but also for safeguarding valuable genetic resources within this culturally and agriculturally important genus.

##### Discussion.

The discovery of *Colocasia sookchaloemiae* highlights the underestimated diversity of the genus in Thailand. Until recently, the Thai flora recognized only a limited number of *Colocasia* species (three, i.e., *C. esculenta*, *C. fallax*, and *C. menglaensis*; Boyce and Sookchaloem 2012). The addition of *C. sookchaloemiae* demonstrates that northeastern Thailand, particularly protected areas such as Phu Pha Yol National Park, harbors cryptic taxa awaiting discovery. This finding is consistent with recent studies of Araceae from the region, including *Alocasia sakonakhonensis* and *Amorphophallus sakonnakhonensis* (Promprom et al. 2023; Promprom et al. 2024), which collectively reinforce the importance of this area as a regional center of aroid diversity.

The recognition of *Colocasia sookchaloemiae* also contributes to broader discussions of species delimitation within *Colocasia*. As noted by Sangnin and Sookchaloem (2008), morphological variability in Thai populations often masks cryptic diversity, and the new species exemplifies how traits such as dormancy, stigma form, and interstice indumentum can provide diagnostic value. These findings support the view that the genus in mainland Southeast Asia remains incompletely understood, with further field and herbarium studies likely to yield additional taxa.

From an ecological perspective, *Colocasia sookchaloemiae* occupies rocky habitats within dry dipterocarp and mixed deciduous forests at mid elevations (350–400 m a.s.l.). This contrasts with the typically moist habitats of many congeners, such as *C. esculenta*, *C. fallax*, and *C. menglaensis*, which commonly occur in moist to wet environments (Mayo et al. 1997; Boyce and Sookchaloem 2012), suggesting ecological specialization. Such adaptation may have driven morphological divergence, as proposed for other narrowly distributed Araceae in seasonal habitats. Its occurrence in at least two populations across provincial boundaries implies a potentially wider, though still restricted, distribution within the protected landscape of Phu Pha Yol National Park. However, its confinement to a limited area and apparent habitat specificity warrant conservation attention.

In summary, *Colocasia sookchaloemiae* enriches the taxonomy of *Colocasia* in Thailand and underlines the floristic uniqueness of Phu Pha Yol National Park. Its clear morphological distinction from *C. fallax* and related taxa supports its recognition as a new species, while its restricted distribution underscores the importance of continued exploration and conservation in the understudied ecosystems of northeastern Thailand.

##### Additional examined specimens (Paratype).

Thailand • Sakon Nakhon, 31 August 2023, alt. ca. 400 m, W. Chatan 2473 (paratype: BKF).

## Supplementary Material

XML Treatment for Colocasia sookchaloemiae

## ﻿Additional information

### Use of AI

AI was used only to improve the English language and did not contribute to the scientific content.

### Author ORCIDs

Wilawan Promprom https://orcid.org/0000-0001-8821-2267

Phukphon Munglue https://orcid.org/0000-0001-7247-2873

Pattana Pasorn https://orcid.org/0009-0007-3808-7331

Soulivanh Lanorsavanh https://orcid.org/0000-0002-8433-7297

Wannachai Chatan https://orcid.org/0000-0002-3941-7626

### Data availability

All of the data that support the findings of this study are available in the main text.
